# A fair and EMG-validated comparison of recruitment criteria, musculotendon models and muscle coordination strategies, for the inverse-dynamics based optimization of muscle forces during gait

**DOI:** 10.1186/s12984-021-00806-6

**Published:** 2021-01-28

**Authors:** Florian Michaud, Mario Lamas, Urbano Lugrís, Javier Cuadrado

**Affiliations:** Laboratory of Mechanical Engineering, University of La Coruña, Ferrol, Spain

**Keywords:** Static optimization, Musculotendon models, Gait, Muscle forces, EMG validation

## Abstract

Experimental studies and EMG collections suggest that a specific strategy of muscle coordination is chosen by the central nervous system to perform a given motor task. A popular mathematical approach for solving the muscle recruitment problem is optimization. Optimization-based methods minimize or maximize some criterion (objective function or cost function) which reflects the mechanism used by the central nervous system to recruit muscles for the movement considered. The proper cost function is not known a priori, so the adequacy of the chosen function must be validated according to the obtained results. In addition of the many criteria proposed, several physiological representations of the musculotendon actuator dynamics (that prescribe constraints for the forces) along with different musculoskeletal models can be found in the literature, which hinders the selection of the best neuromusculotendon model for each application. Seeking to provide a fair base for comparison, this study measures the efficiency and accuracy of: (i) four different criteria within the static optimization approach (where the physiological character of the muscle, which affects the constraints of the forces, is not considered); (ii) three physiological representations of the musculotendon actuator dynamics: activation dynamics with elastic tendon, simplified activation dynamics with rigid tendon and rigid tendon without activation dynamics; (iii) a synergy-based method; all of them within the framework of inverse-dynamics based optimization. Motion/force/EMG gait analyses were performed on ten healthy subjects. A musculoskeletal model of the right leg actuated by 43 Hill-type muscles was scaled to each subject and used to calculate joint moments, musculotendon kinematics and moment arms. Muscle activations were then estimated using the different approaches, and these estimates were compared with EMG measurements. Although no significant differences were obtained with all the methods at statistical level, it must be pointed out that a higher complexity of the method does not guarantee better results, as the best correlations with experimental values were obtained with two simplified approaches: the static optimization and the physiological approach with simplified activation dynamics and rigid tendon, both using the sum of the squares of muscle forces as objective function.

## Introduction

Determination of muscle forces during gait is of great interest to extract the principles of the central nervous system (CNS) control, to facilitate assessment of pathological gait, or to estimate the loads on bones and joints (prevention of injuries in sports, surgical planning to reconstruct diseased joints) [1–3]. The invasive character of in vivo experimental measurements, and the uncertain relation between muscle force and EMG, makes computer modeling and simulation a useful substitutive approach. Determination of muscle forces by computer modeling and simulation was extensively treated and numerous approaches can be found in the literature to solve the redundancy problem of the muscle recruitment, as well as to represent the musculotendon actuator dynamics [4–8], where each author highlights the advantages of his own approach. Ambrosio and Kecskemethy [4] suggested that the physiological criteria improve human motion prediction, Hardt [5] defended the linear constraint programming, Anderson and Pandy [6] showed that static and dynamic optimization solutions for gait are practically equivalent, Millard et al. [7] offered a damped equilibrium musculotendon model to improve the physiological optimization, and Shourijeh et al. [8] found that forward static optimization was a suitable method for solving forward dynamic musculoskeletal simulations. However, results do not depend only on the approach used, but also, on the experimental data collection and on the musculoskeletal model used, which makes more difficult for the readers to select objectively which approach to use for a certain application [9].

To objectively compare different approaches, it is necessary to test them under the same conditions. When proposing a new approach, authors generally make a comparison with experimental measurements in order to validate it [10, 11], and, in some cases, they compare their results with those provided by a previous approach which gives confidence to readers [12, 13]. In some applications, a benchmark problem can be found that establishes some defined conditions, so that researchers can get a fair comparison [14]. In the case of the resolution of the muscle force-sharing problem, the authors only found a benchmark where the computational speed and biological accuracy of three musculotendon models was compared during simple muscle-driven simulations [7]. The physiological effect of static and dynamic optimization during gait was compared by Pandy et al. [10] and De Groote et al. [15], but none of the studies offered an experimental validation that allowed to conclude which method provided the most realistic results.

Few years ago, a grand challenge competition to predict in vivo knee loads was organized by some researchers who shared their experimental data collections for the analysis and its evaluation [16]. However, the musculoskeletal modeling could differ between participants so that, by using a different multibody model (with different degrees of freedom) and different muscle geometry (which implies different arm moments), the results could not dissociate the effect of neuromusculotendon models.

In this context, the aim of the present work is to compare the efficiency and accuracy of: i) four different cost functions within the static optimization approach (where the physiological character of the muscle, which affects the constraints of the forces, is not considered); ii) one static and three physiological representations of the musculotendon actuator dynamics: activation dynamics with elastic tendon, simplified activation dynamics with rigid tendon, and rigid tendon without activation dynamics; iii) a synergy-based method; all of them within the framework of inverse-dynamics based optimization. The best cost function (criterion) obtained in (i) was used for (ii) and (iii). Motion/force/EMG gait analyses were performed on ten healthy subjects. A musculoskeletal model of the right leg actuated by 43 Hill-type muscles was scaled to each subject and used to calculate joint moments, musculotendon kinematics and moment arms. Therefore, the muscle force-sharing problem was solved under the same conditions and using the same inputs. Muscle activations were then estimated using the different approaches, and these estimates were compared with EMG measurements which served as experimental reference.

## Methods

### Experimental data collection

**Fig. 1:**
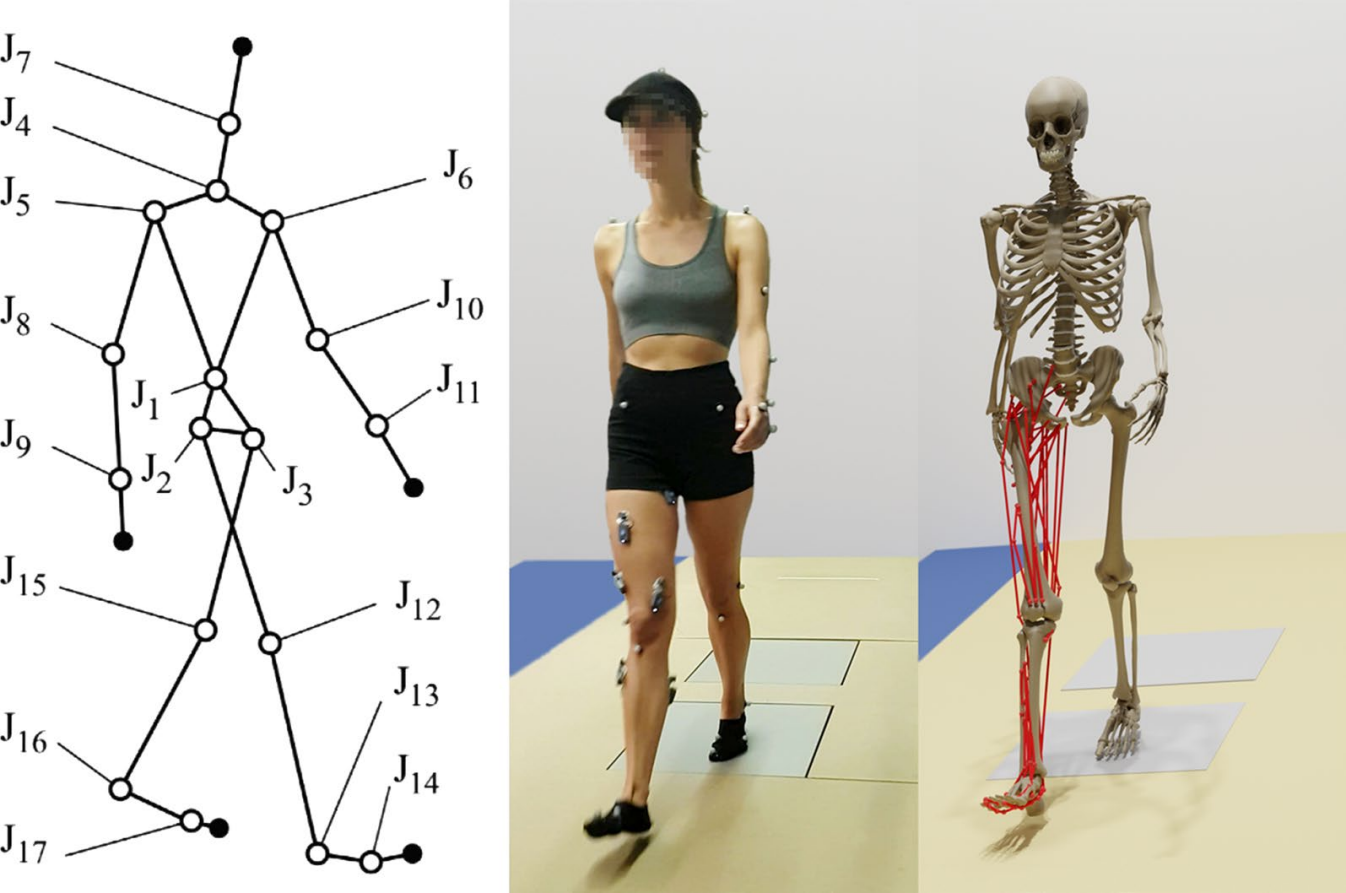
Gait of healthy subject: multibody model (left); acquired motion (middle); computational model (right)

Ten subjects (seven males, three females, age 42 ± 16 years, height 173 ± 16 cm, body mass 73 ± 26 kg) were recruited for this study. All subjects gave written informed consent for their participation. Subjects walked at their self-selected speed (1.1 ± 0.2 m/s) along a walkway with two embedded force plates (AccuGait, sampling at 100 Hz; AMTI, Watertown, MA, USA). The motion was captured using 12 optical infrared cameras (OptiTrack FLEX 3, also sampling at 100 Hz; Natural Point, Corvallis, OR, USA) that computed the position of 36 optical markers (Fig. 1). An extended Kalman filter (EKF) was used to filter the marker trajectories and reconstruct the motion with a process noise variance of 1 m/s2 and a cutoff frequency of 20 Hz [17]. Additionally, 9 surface EMG signals were recorded from the right leg at 1 kHz (BTS, FREEEMG, Quincy, MA, USA). The participants' right leg was shaved, the skin was cleaned with alcohol, and the electrodes were placed according to the guideline presented in [18]. EMG signals were acquired from the following muscles: tibialis anterior, vastus medialis, vastus lateralis, gastrocnemius medialis, gastrocnemius lateralis, semitendinosus, biceps femoris, gluteus maximus and gluteus medius. Each EMG signal was rectified, filtered by singular spectrum analysis (SSA) with a window length of 250 [19] (equivalent to the common forward and reverse low-pass 5th order Butterworth filter with a cut-off frequency of 6 Hz), and, then, normalized with respect to its maximal value. This cut-off frequency value is consistent with the ranges reported in previous lower extremity studies using EMG data [20–24].

### Musculoskeletal model

The human body was modeled as a three-dimensional multibody system formed by rigid bodies (Fig. 1, left and center). The model consisted of 18 anatomical segments [25]: two hindfeet, two forefeet, two shanks, two thighs, a pelvis, a torso, a neck, a head, two arms, two forearms, and two hands. The segments were linked by ideal spherical joints, thus defining a model with 57 degrees of freedom (DOFs). The axes of the global reference frame were defined as follows: *x*-axis in the anterior–posterior direction, *y*-axis in the medial–lateral direction, and *z*-axis in the vertical direction. The computational model was defined with 228 mixed (natural + angular) coordinates. The subset of natural coordinates comprised the three Cartesian coordinates of 22 points and the three Cartesian components of 36 unit vectors, thus yielding a total of 174 variables.

Matrix-R formulation [26] was used to perform an inverse-dynamics analysis to obtain the joint torques along the motion by means of the in-house developed MBSLIM library [27] programmed in FORTRAN, as described in [28]. Once the joint torques were computed, it was assumed that 43 right leg muscles contributed to the following six right-leg inverse-dynamics moments: the three rotational DOFs at the hip, the flexion/extension DOF at the knee, and the plantar/dorsi flexion and inversion/eversion at the ankle. Muscles were modeled as one or more straight-line segments with via points. These points corresponded to the attachments of muscle and tendon to bone and were defined as the origin (i.e., proximal attachment) and insertion (i.e., distal attachment). Muscle properties and local coordinates for these points were obtained from OpenSim (model Gait2392) [29] and scaled to each subject from the generic reference OpenSim model, as commented further in 2.4.

### Optimization problem

As introduced before, the fundamental problem is that there are more muscles serving each degree of freedom of the system than those strictly necessary from the mechanical point of view. In this case, there are 43 muscles at the leg to actuate 6 degrees of freedom (other degrees of freedom of the leg are controlled by joint structures as bones and ligaments, yielding a reaction moment instead a drive torque). Consequently, there is an infinite number of solutions for this problem and, in order to reproduce the specific strategy of muscle coordination adopted by the CNS, optimization is used.

The inverse-dynamics based optimization problem that serves to determine the muscle forces at each time-point can be formulated in general form as:

1 $$\begin{gathered} \min \, C \hfill \\ {\text{subject to }}{\mathbf{J}}^{T} {\mathbf{F}}^{MT} = {\mathbf{Q}}^{ID} \hfill \\ \, F_{i}^{Min} \le F_{i}^{MT} \le F_{i}^{Max} \, i = 1,2,...,m \hfill \\ \end{gathered}$$

where $C$ is the cost function, ${\mathbf{Q}}^{ID}$ is the vector of inverse-dynamics joint moments at the right leg (where the force-sharing problem is addressed), ${\mathbf{F}}^{MT}$ is the vector of muscle forces, ${\mathbf{J}}$ is the Jacobian whose transpose projects the muscle forces into the joint drive torques space, $F_{i}^{Min}$ and $F_{i}^{Max}$ are the instantaneous minimum and maximum allowed forces in muscle *i*, respectively, and *m* is the number of muscles. Expression of the objective function *C* depends on the muscle recruitment criterion used. In the literature, several muscle recruitment criteria have been suggested to represent the CNS behavior. In this work, four of them have been considered in the context of static optimization.

#### Static optimization (SO)

##### Nonlinear polynomial criteria

The polynomial criterion can be written as

2 $$\min \sum\limits_{i = 1}^{m} {\left( {\frac{{F_{i}^{MT} }}{{k_{i} }}} \right)}^{w}$$

where $k_{i}$ denotes a positive weighting factor and $w$ is the power of the polynomial. According to [30], the muscle force prediction obtained by minimizing the sum of muscle stresses raised to a power *w* whose value ranges between 1.4 and 5.1 is physiologically analogous to minimizing muscle fatigue. As Anderson and Pandy did in their study [10], a power of 2 was chosen.

##### Criterion I—minimization of the sum of the squares of muscle forces

3 $$\min \sum\limits_{i = 1}^{m} {\left( {F_{i}^{MT} } \right)}^{2} ;$$

##### Criterion II—minimization of the sum of the squares of relative muscle forces

4 $$\min \sum\limits_{i = 1}^{m} {\left( {\frac{{F_{i}^{MT} }}{{F_{0,i}^{M} }}} \right)}^{2} ;$$

with $F_{0}^{M}$ the maximum isometric force from [29].

##### Criterion III—minimization of the sum of the squares of muscle stresses

5 $$\min \sum\limits_{i = 1}^{m} {\left( {\frac{{F_{i}^{MT} }}{{PCSA_{i} }}} \right)}^{2} ;$$

with $PCSA$ the physiological cross sectional area from [31].

##### Min/max criterion

The min/max criterion distributes the collaborative muscle forces in such a way that the maximum relative muscle force is as small as possible. Therefore, the largest endurance for a task is attained when the maximum relative muscle force [32] or the maximum muscle stress [33] is as small as possible. The min/max criterion takes the form:

6 $$\min \left( {\max \left( {\frac{{F_{i}^{MT} }}{{k_{i} }}} \right)} \right),i = 1,...,m;$$

For this study, the following criterion is used:

##### Criterion IV—minimization of the largest relative muscle force

7 $$\min \left( {\max \left( {\frac{{F_{i}^{MT} }}{{F_{0,i}^{M} }}} \right)} \right),i = 1,...,m;$$

For SO, the physiological behavior of the musculotendon actuator dynamics is not considered, so, the limit values of the muscular forces are $F_{i}^{Min} = 0$ and $F_{i}^{Max} = F_{0,i}^{M}$.

##### Physiological approach (PHY1)

**Fig. 2:**
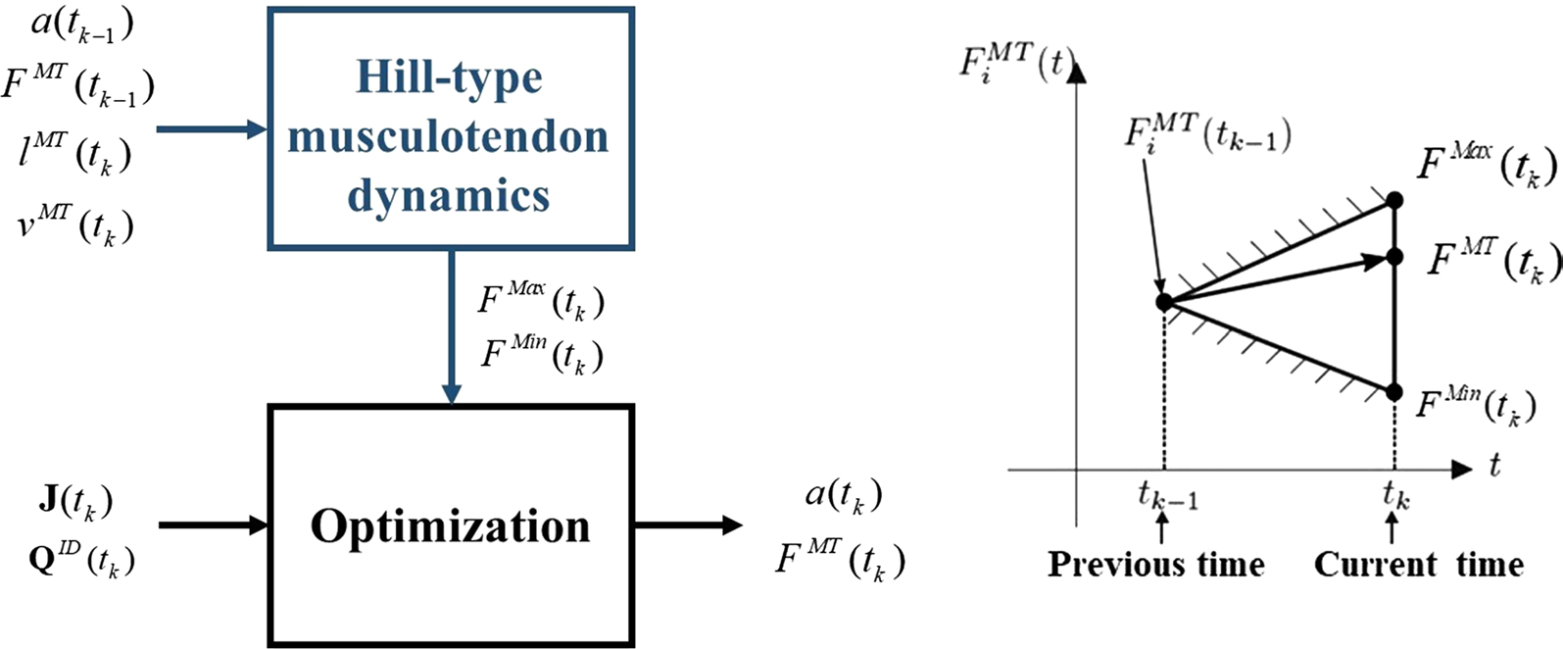
Procedure using physiological inverse-dynamics approach to determine individual muscle forces at time instant *t*_*k*_ (left); Minimal and maximal constraints for a muscle force by extrapolation of the force value from the previous time-point (right)

At physiological level, musculotendon actuator dynamics introduces muscle force constraints. Whereas the static optimization approach disregards these constraints in order to simplify the problem, the so-called physiological approach [34] takes them into consideration. This approach applies optimization techniques at each time-point (Fig. 2, right), and prescribes minimal and maximal constraints for the forces by extrapolating the force values from the previous time-point through feasible muscle dynamics (Fig. 2, left).

The dynamics of musculotendon actuators can be divided into two parts. First, the activation dynamics which corresponds to the transformation of a neural excitation sent by the brain into an activation of the contractile apparatus. Activation dynamics is described by a first-order ordinary differential equation that contains the relationship among the muscle activation $a$, its derivative $\dot{a}$, and the neural excitation $u$ as:

8 $$\dot{a}(t) = \frac{u(t) - a(t)}{\tau },$$

with $\tau = \tau_{act}$ when $a(t_{k - 1} ) \le u(t_{k} )$ and $\tau = \tau_{deact}$ when $a(t_{k - 1} ) > u(t_{k} )$. The activation and deactivation time constants $\tau_{act}$ and $\tau_{deact}$ are set to 15 ms and 50 ms, respectively [35, 36].

**Fig. 3:**
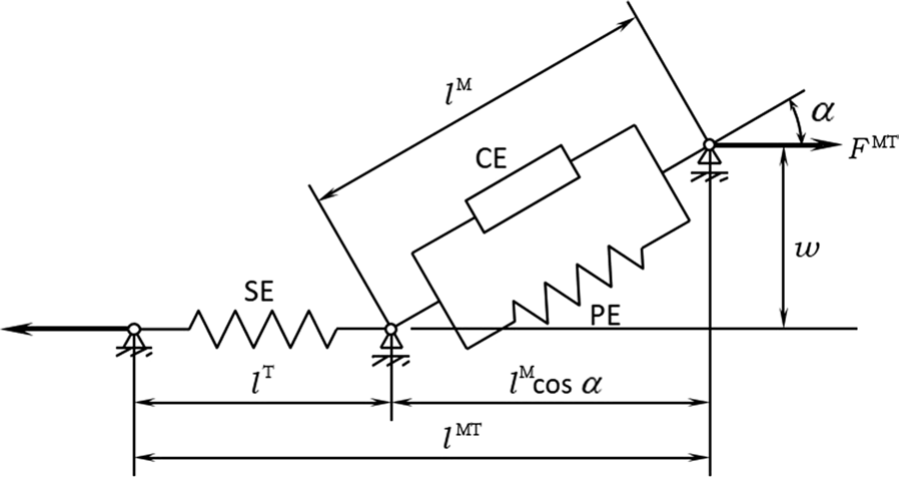
Hill-type muscle model. The muscle fibers are modeled as an active contractile element (CE) in parallel with a passive elastic component (PE). These elements are in series with a nonlinear elastic tendon (SE). The pennation angle denotes the angle between the muscle fibers and the tendon. Superscripts MT, M, and T indicate musculotendon, muscle fiber, and tendon, respectively

Second, this activation is transformed into a muscle force by the second phase, the contraction dynamics. The force generated by a muscle is constrained by its force–length-velocity properties, related to the Hill-type musculotendon model used (Fig. 3), which is defined by this second differential equation:

9 $$\dot{F}^{MT} (t) = f(a(t),F^{MT} (t),l^{MT} (t),v^{MT} (t)).$$

The musculotendon length $l^{MT}$ and velocity $v^{MT}$ depend on the position and velocity of the body segments and, in turn, the generated tendon force $F^{MT}$ affects the motion of the body segments. Thus, there exists interaction between muscles and body segments.

The complete musculotendon dynamics can be expressed as a system of two differential equations which can be written, in a simplified form, as.

10 $$\dot{x}(t) = \left[ {\begin{array}{*{20}c} {\dot{a}(t)} \\ {\dot{F}^{MT} (t)} \\ \end{array} } \right] = h(x(t),u(t),l^{MT} (t),v^{MT} (t)).$$

This system is used to define the minimal and maximal constraints for the forces by extrapolating the force values from the previous time-point using feasible muscle dynamics, integrating (10) with $u(t) = 0$ to estimate $F^{Min} (t)$ and with $u(t) = 1$ to estimate $F^{Max} (t)$ (the muscular excitation is assumed to be constant between the two time frames). Using the physiological approach, the initial activations and muscles forces are needed. The determination of initial activations and muscular forces is based on the static condition which states that the initial fiber velocity of each muscle is set to zero and, $F^{Min}$ and $F^{Max}$ correspond to $a = 0$ and $a = 1$, respectively.

Integration is carried out with Matlab *Ode23t*. Two integrations per muscle are required at each time step, which make the optimization and integration process heavy and slow. By programming the muscular functions of the Hill-type musculotendon model into a FORTRAN mex file, the computational time is reduced by a factor of 10. However, the computational time is still long and, in addition, the high tendon stiffness makes really difficult to use this approach (a suitable scaling of muscle parameters is needed) [37]. Therefore, in order to simplify the problem while keeping some physiological characteristics, most authors prefer to use a Hill-type musculotendon model with a rigid tendon [13, 15, 38].

### Physiological approach with rigid tendon

In this way, the tendon length is constant and, consequently, the muscle fiber length and velocity depend only on the musculoskeletal geometry as well as on body segment configurations (which affect $l^{MT}$ and $v^{MT}$) and not the musculotendon force. Consequently, the force–length-velocity allowed is expressed as:

11 $$F^{MT} (t) = a(t)g\left( {l^{MT} (t),v^{MT} (t)} \right).$$

Use of the rigid tendon model avoids the two integrations needed to calculate the limits of the muscle force at each instant. Then, in order to further reduce the computational burden, the first-order ordinary differential Eq. (8) used to estimate the muscular activation, $a$, can be simplified as follows:

### Time response considered (PHY2)

In order to keep the muscular time response relation given by (8), the first-order ordinary differential equation can be converted into:

12 $$a(t_{k} ) = u(t_{k} ) + (a(t_{k - 1} ) - u(t_{k} ))e^{( - \Delta t/\tau )} ,$$

with $\tau = \tau_{act}$ when $a(t_{k - 1} ) \le u(t_{k} )$, $\tau = \tau_{deact}$ when $a(t_{k - 1} ) > u(t_{k} )$ and $\Delta t$ is the time step.

Therefore, the minimal and maximal muscular force constraints of the optimization problem can be obtained through integration with any of the described approaches by extrapolating the force values from the previous time-point using feasible muscle dynamics.

### Time response ignored (PHY3)

However, authors who consider the tendon as a rigid element usually choose to ignore the muscular time response and assume that:

13 $$a(t) = u(t).$$

In this work, the minimization of the sum of the squares of muscle forces (Criterion I) was used as objective function for the three physiological models.

#### Synergy optimization (SynO)

The fact that synergies take a high dimensional control space and reduce it to a low dimensional space is potentially useful for reducing the amount of indeterminacy when estimating muscle forces via optimization. For this reason, some authors started to investigate how to include it to solve the muscle force-sharing problem.

The synergy optimization (SynO) approach used in [13] estimates muscle forces during human walking using synergy-constructed muscle activations, similar to the more complex approach proposed in [39]. SynO finds muscle forces that match the inverse-dynamics joint moments as closely as possible through the moment tracking error term in the cost function. In SynO, synergies couple muscle activations across time frames, requiring the optimization to be performed over all the time frames simultaneously as follows:

14 $$a^{*}_{{f \times {\text{m}}}} = T_{{f \times n_{S} }} (T_{p} )V_{{n_{S} \times m}} \,$$

where $T_{{f \times n_{S} }} (T_{p} )$ and $V_{{n_{S} \times m}} \,$ are the time-varying synergy activations defined by B-spline nodes, and the corresponding time-invariant synergy vectors, respectively. Each muscle activation synergy is composed of a single time-varying synergy activation defined by *p* = *(f-1)/5* + *1* (nearest integer, *f* = number of frames) B-spline nodal points along with its corresponding time-invariant synergy vector defined by *m* = 43 weights specifying inter-muscle activation coupling. Thus, for *n*_*S*_ synergies (*n*_*S*_ = 3 in this study), the number of design variables is *n*_S_*(p + *m)*. Muscle synergy quantities are used as the design variables for synergy optimization. Each optimization problem is theoretically over-determined. However, in practice, the problems remain under-determined since neighboring time frames are not completely independent from one another.

Using these design variables, the SynO cost function is formulated as follows:

15 $$\mathop {C_{SynO} }\limits_{{C_{p} ,V}} = \sum\limits_{j = 1}^{n} {\left( {\beta \sum\limits_{k = 1}^{6} {\left[ {\frac{{Q_{jk}^{MT} - Q_{jk}^{ID} }}{{\max (|Q_{k}^{ID} |)}}} \right]}^{2} + \sum\limits_{i = 1}^{m} {\left( {a_{ij}^{*2} + \lambda_{ij,pen} (a_{ij}^{*} - 1)^{2} } \right)} } \right)}$$

where $a_{ij}^{*}$ are the synergy-based muscle activations, and $$\lambda_{ij,pen} = \left\{ \begin{gathered} 0{ 0} \le a^{*}_{ij} \le 1 \hfill \\ 10^{5} {\text{ otherwise}} \hfill \\ \end{gathered} \right.$$ are penalization factors for muscle *i* at the time frame *j* to ensure that muscle activations stay between 0 and 1. While previous approaches enforce the muscle forces to exactly reproduce the inverse-dynamics joint moments through its equality constraints, the SynO approach minimizes the error between $Q_{{}}^{ID}$ and $Q_{{}}^{MT}$, being $Q_{{}}^{MT}$ the joint moments produced by the muscle forces estimated by SynO. A scale factor $\beta = 100$ is applied to achieve the best compromise between joint moment tracking and activation minimization [40]. In his previous work [40], Michaud found that best correlations with experimental EMG patterns were obtained using three synergies for SynO, and that the resulting mean joint intersegmental moment matching between $Q_{{}}^{ID}$ and $Q_{{}}^{MT}$ across subjects was higher than 96%. For this reason, the SynO approach will be used in this study with only three synergies.

The objective function is programmed as a FORTRAN mex file to reduce computation time (16 times faster than the original Matlab function). Linear equality constraints enforce that the sum of weights within each synergy vector is equal to 1, which makes the synergy construction unique, while lower bound constraints enforce the synergy activation B-spline nodes and synergy vector weights to be non-negative. The same musculotendon model used for the PHY3 approach is used here.

#### Subject-specific scaling of musculotendon parameters

Due to the sensitivity of physiological approaches [37], a suitable scaling of musculotendon parameters is needed. In addition to the high tendon stiffness which makes implementation really difficult, some Hill-muscle equations become numerically stiff when numerical singularities are approached [7]. Since these conditions are often encountered during a simulation, to prevent that the solver gets stuck at points that were numerically feasible yet not physiologically sound [41] (which slows the process of numerical integration), a scaling correction was applied. Length parameters were scaled in two steps. First, for each muscle, the tendon slack length ($l_{S}^{T}$) and the optimal muscle fiber length ($l_{0}^{M}$) were scaled with a scale factor calculated as the relation between the subject’s musculotendon length in standing position and that of the generic model in the same position. As the pennation angle of the reference, $\alpha_{0}$, is kept, the scaled distance between the aponeuroses of muscle origin and insertion, $w$ (which remains constant during the muscle contraction), is given by:

16 $$w = l^{M} \sin (\alpha ) = l_{0}^{M} \sin (\alpha_{0} ).$$

Then, because tendons are so stiff that their lengths do not change significantly during movement, the approximated muscle fiber length, $l^{{M*}}$, can be calculated for each muscle during the complete gait cycle as follows:

17 $$l^{{M*}} (t) = {{{\left( {l^{{MT}} (t) - l_{S}^{T} } \right)} \mathord{\left/ {\vphantom {{\left( {l^{{MT}} (t) - l_{S}^{T} } \right)} {\cos (\alpha (t)}}} \right. \kern-\nulldelimiterspace} {\cos (\alpha (t))}}}$$

with $\alpha (t) = \arctan \left( {\frac{w}{{l_{{}}^{MT} (t) - l_{S}^{T} }}} \right)$. Finally, in order to keep the normalized muscle lengths ($\overline{{l_{{}}^{M} }} = \frac{{l^{M} }}{{l_{0}^{M} }}$) within the physiological optimal conditions ($0.5 < \overline{{l^{M} }} \le 1.2$) [42], the final scaled $l_{0}^{M}$ was set to the maximum approximated muscle fiber length along the motion.

### Optimization protocol and EMG comparison

Optimization seeks to find the best solution from all the feasible ones by minimizing the objective function. Finding the global minima of a function is really difficult because of the many local minima. In order to get the best possible results, the following protocol is used in this work. Although each optimization problem is solved using the Matlab’s *fmincon* nonlinear constrained optimization algorithm, five global optimizations are run using Matlab’s *ga* genetic optimization algorithm with a population size of 50 to provide random initial guesses for *fmincon*. The solution with the lowest objective function value is chosen as initial guess for the initial time point. Thereafter, as muscle activation is normally smooth and continuous during gait, the optimal solution from the previous time frame is used as the initial guess for the current time frame [43].

Matching between estimated muscle activations and EMG was quantified via cross-correlation using the Pearson correlation coefficient *r* (Matlab’s function *corrcoef*) with a maximum time delay of 150 ms [44]. The correlation coefficient *r* was chosen to compare muscle activations and EMG data so as to focus on shape rather than on magnitude discrepancies, as there is no direct relationship between EMG and muscle force amplitude [45, 46].

## Results

**Fig. 4:**
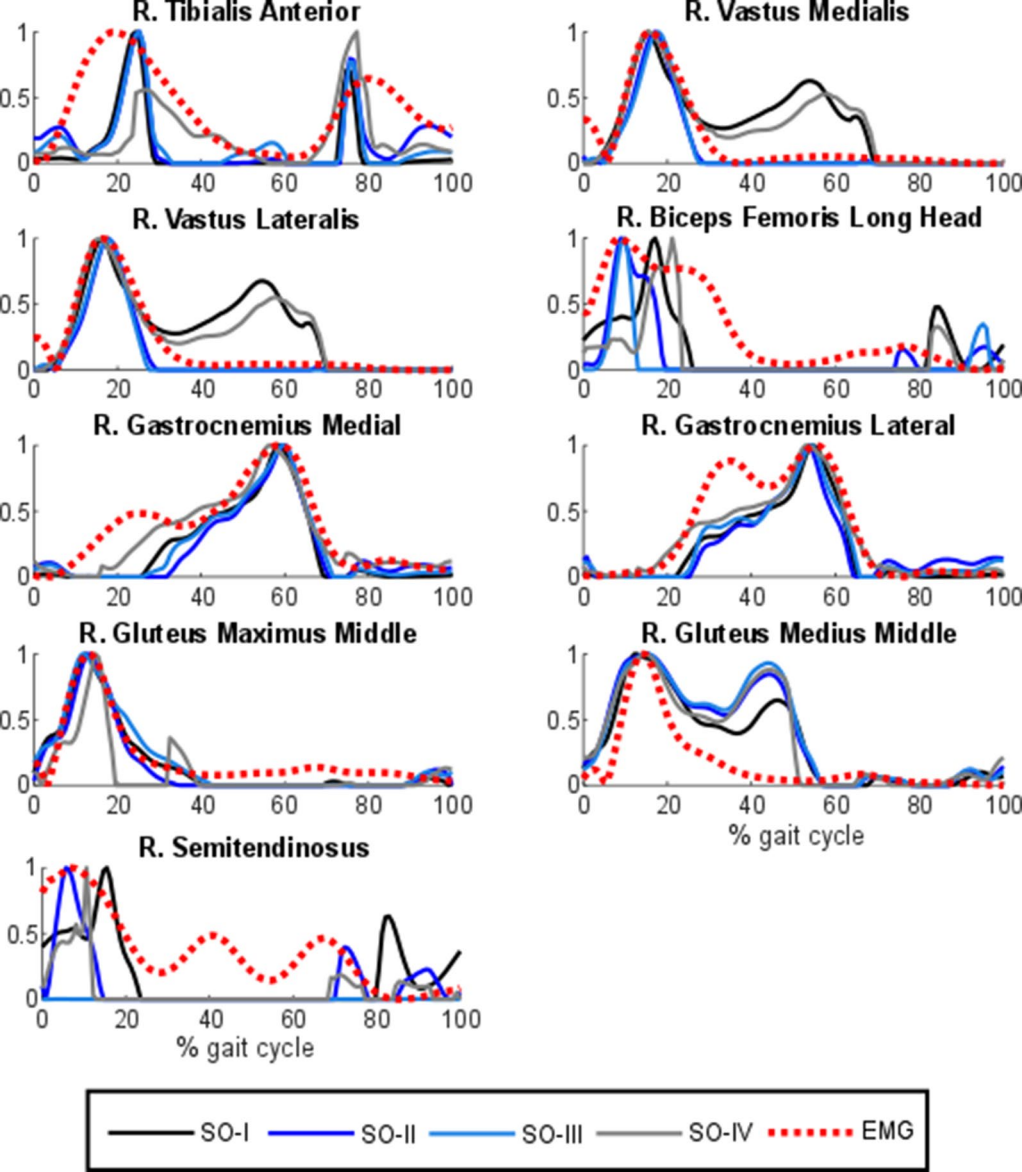
Normalized muscular activations obtained with static optimization (criteria I-IV) vs. normalized EMG for a healthy subject

The different approaches presented in this study were compared with EMG measurements for the ten healthy subjects. Normalized muscle activations during a gait cycle of one healthy subject estimated by SO using all the criteria are plotted in Fig. 4 along with the corresponding normalized EMG measurements. Comparison of muscle activations estimated with the different criteria are significantly different but show some similarities too.

**Fig. 5:**
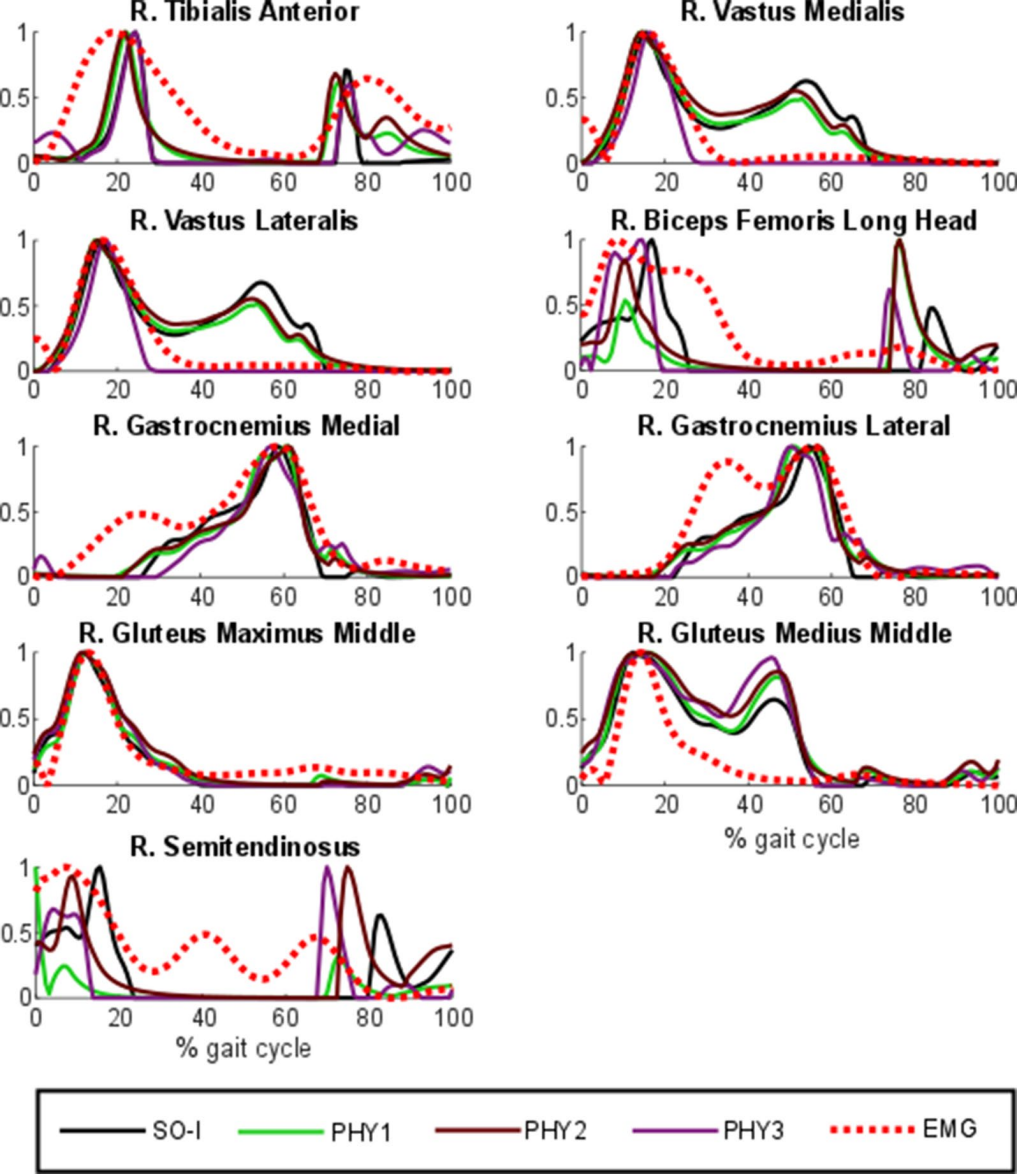
Normalized muscular activations obtained through static optimization with criterion I (SO-I) and physiological optimization with criterion I (original approach and two simplified alternatives) vs. normalized EMG for a healthy subject. PHY1: physiological approach; PHY2: physiological approach with rigid tendon and activation time response considered; PHY3: physiological approach with rigid tendon and activation time response ignored

Then, to observe the physiological effect of the musculotendon model, four approaches using criterion I were compared: normalized muscle activations during a gait cycle of one healthy subject estimated by static optimization (SO-I) and the three physiological approaches (PHY1, PHY2 and PHY3) are represented in Fig. 5. While PHY3 presented distinct results, muscle activations estimated by SO-I, PHY1 and PHY2 were very similar. PHY1 and PHY2 showed almost the same results.

**Fig. 6:**
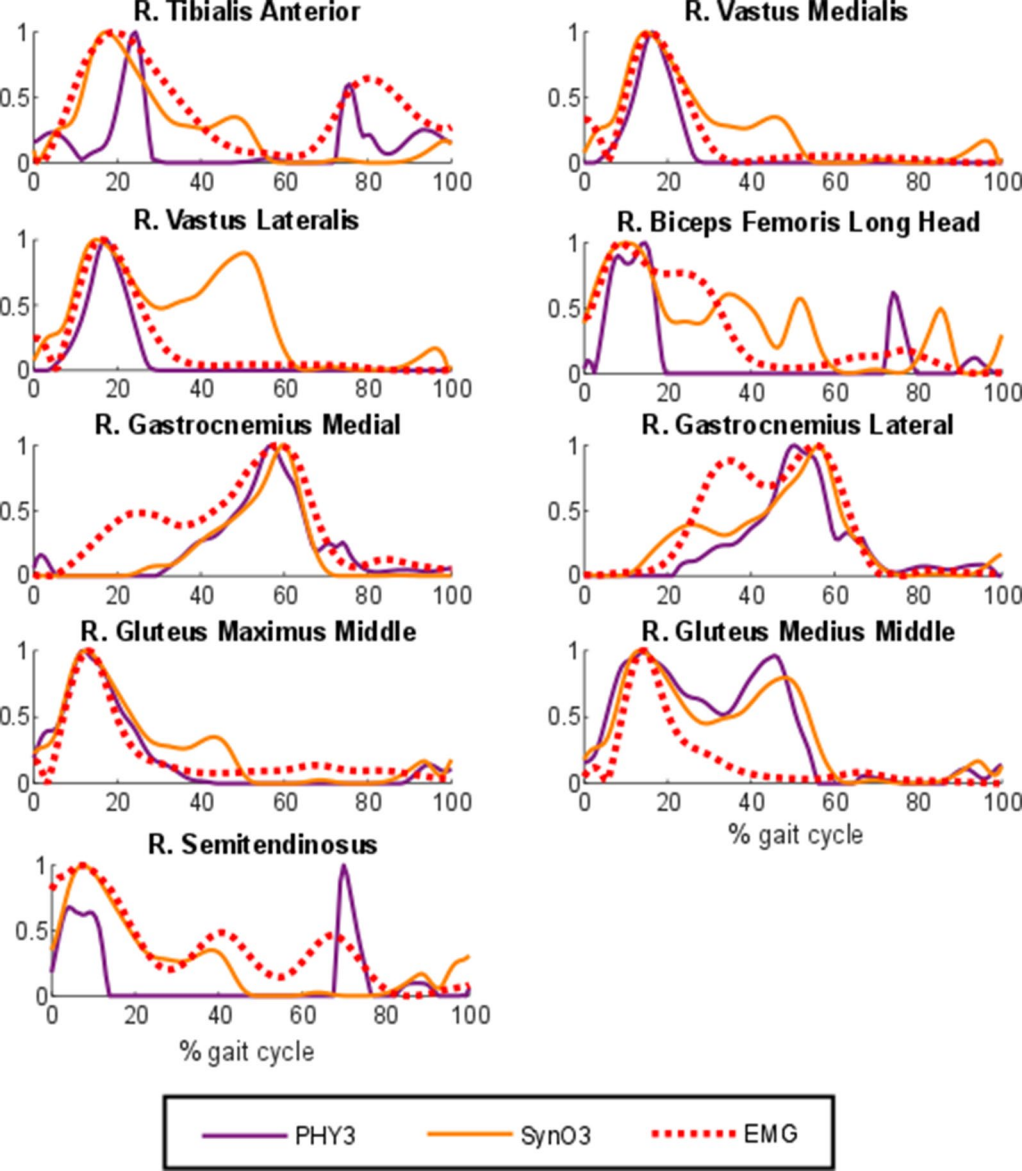
Normalized muscular activations obtained from physiological optimization with rigid tendon and activation time response ignored (PHY2) and synergy optimization with 3 synergies (SynO3) vs. normalized EMG for a healthy subject

Furthermore, the normalized muscle activations during a gait cycle of one healthy subject estimated by PHY3 and synergy optimization with 3 synergies (SynO3) are compared in Fig. 6 to highlight the effect of the synergy structure. Both used the same musculotendon model (physiological approach with rigid tendon and activation time response ignored). However, results are significantly different.

Mean across subjects Pearson correlation coefficient *r* values between EMG vs. muscle activations of all the approaches of this study for the nine muscles are reported in Table 1. Mean correlations of the many approaches did not present as significant differences as those observed previously for one subject. Mean values of the different approaches are close, between 0.61 (SO-IV and SynO3) and 0.74 (SO-I and PHY2). Approaches SO-I, PHY1 and PHY2 show almost the same correlations (means of 0.73 and 0.74). The paired sampled t-test realized (Table 2) showed no statistical differences (p < 0.05) between SO-I, SO-II, PHY1, PHY2 and PHY3, while differences were observed with the other approaches.

**Table 1:** Mean across subjects Pearson correlation coefficient r values between EMG vs. muscle activations (r < 0.40 underlined and r > 0.60 in italics) and computational time of the different approaches

	Mean values							
	Pearson correlation coefficient r between across-subjects mean EMG vs. muscle activations							
	SO-I	SO-II	SO-III	SO-IV	PHY1	PHY2	**PHY3**	**Syn03**
R. Tibialis Anterior	*0.61*	*0.65*	0.52	0.23	*0.62*	*0.65*	*0.67*	0.37
R. Vastus Medialis	*0.68*	*0.67*	*0.67*	0.55	*0.79*	*0.79*	*0.75*	*0.73*
R. Vastus Lateralis	*0.68*	*0.70*	*0.70*	*0.63*	*0.79*	*0.79*	*0.82*	*0.66*
R. Gastrocnemius Medial	*0.86*	*0.76*	*0.84*	*0.80*	*0.79*	*0.80*	*0.76*	*0.85*
R. Gastrocnemius Lateral	*0.75*	*0.67*	*0.73*	*0.65*	*0.69*	*0.70*	*0.67*	*0.74*
R. Semitendinosus	*0.68*	*0.65*	0.49	0.59	*0.61*	*0.64*	0.58	0.56
R. Biceps Femoris Long Head	*0.78*	*0.77*	0.59	0.56	*0.70*	*0.74*	*0.65*	0.37
R. Gluteus Maximus Middle	*0.89*	*0.82*	*0.87*	*0.81*	*0.89*	*0.88*	*0.86*	*0.66*
R. Gluteus Medius Middle	*0.71*	*0.63*	*0.61*	*0.62*	*0.69*	*0.69*	0.56	0.58
Mean	*0.74*	*0.71*	*0.67*	*0.61*	*0.73*	*0.74*	*0.70*	*0.61*
Mean computational time	2.5	27.6	16.1	19.2	225.2	3.6	3.2	48.7

**Table 2:** p-value for paired sample t-test for the Pearson correlation coefficient r between across-subjects mean (p < 0.05 underlined, NA: not applicable)

	P-value for paired sample t-test for the Pearson correlation coefficient r between across-subjects mean							
	**SO-I**	**SO-II**	**SO-III**	**SO-IV**	**PHY1**	**PHY2**	**PHY3**	**Syn03**
SO-I	NA	0.062	0.030	0.005	0.761	0.846	0.327	0.035
SO-II	0.062	NA	0.340	0.071	0.229	0.038	1.000	0.148
SO-III	0.030	0.340	NA	0.104	0.026	0.019	0.272	0.171
SO-IV	0.005	0.071	0.104	NA	0.017	0.013	0.101	0.836
PHY1	0.761	0.229	0.026	0.017	NA	0.065	0.139	0.031
PHY2	0.846	0.038	0.019	0.013	0.065	NA	0.043	0.025
PHY3	0.327	1.000	0.272	0.101	0.139	0.043	NA	0.111
Syn03	0.035	0.148	0.171	0.836	0.031	0.025	0.111	NA

Mean across muscles Pearson correlation coefficient *r* for the ten subjects reported in Table 3 offered similar results as Table 1. Good correlations (*r* > 0.60, in italics) and close results were obtained for most approaches. In Table 4, the paired sampled t-test yielded the same conclusions as Table 2: SO-I, SO-II, PHY1, PHY2 and PHY3 are statistically similar (p < 0.05).

**Table 3:** Mean across muscles Pearson correlation coefficient r values between EMG vs. muscle activations (r > 0.60 in italics)

Subject	Mean Values							
	Pearson correlation coefficient r between across-muscles mean EMG vs. muscle activations							
	**SO-I**	**SO-II**	**SO-III**	**SO-IV**	**PHY1**	**PHY2**	**PHY3**	**Syn03**
1	*0.85*	*0.78*	*0.74*	*0.62*	*0.80*	*0.74*	*0.84*	*0.62*
2	*0.73*	*0.66*	*0.67*	0.50	*0.70*	*0.64*	*0.70*	*0.64*
3	*0.70*	*0.65*	*0.69*	*0.64*	*0.73*	*0.71*	*0.74*	*0.73*
4	*0.74*	*0.78*	*0.72*	*0.72*	*0.71*	*0.76*	*0.72*	*0.70*
5	*0.83*	*0.77*	*0.81*	*0.73*	*0.85*	*0.79*	*0.83*	*0.60*
6	*0.62*	*0.63*	0.56	0.49	*0.65*	*0.68*	*0.65*	*0.63*
7	*0.74*	*0.62*	*0.62*	0.56	*0.66*	*0.65*	*0.74*	0.40
8	*0.71*	*0.67*	0.59	0.53	*0.76*	*0.62*	*0.75*	*0.67*
9	*0.79*	*0.78*	*0.71*	*0.64*	*0.76*	*0.76*	*0.79*	0.53
10	*0.68*	*0.72*	0.60	*0.62*	*0.67*	*0.66*	*0.66*	*0.63*
Mean	*0.74*	*0.71*	*0.67*	*0.61*	*0.73*	*0.70*	*0.74*	*0.61*

**Table 4:** p-value for paired sample t-test for the Pearson correlation coefficient r between across-muscles mean (p < 0.05 underlined, NA: not applicable)

	p-value for paired sample t-test for the Pearson correlation coefficient r between across-muscles mean							
	SO-I	SO-II	SO-III	SO-IV	PHY1	**PHY2**	PHY3	Syn03
SO-I	NA	0.076	0.001	0.000	0.464	0.063	0.718	0.014
SO-II	0.076	NA	0.073	0.000	0.220	0.717	0.090	0.025
SO-III	0.001	0.073	NA	0.004	0.004	0.055	0.001	0.164
SO-IV	0.000	0.000	0.004	NA	0.000	0.000	0.000	0.791
PHY1	0.464	0.220	0.004	0.000	NA	0.138	0.201	0.007
PHY2	0.063	0.717	0.055	0.000	0.138	NA	0.044	0.031
PHY3	0.718	0.090	0.001	0.000	0.201	0.044	NA	0.010
Syn03	0.014	0.025	0.164	0.791	0.007	0.031	0.010	NA

**Table 5:** Mean across subjects of the maximum joint reaction forces at hip, knee and ankle for different muscle recruitment approaches

	**SO-I**	**SO-II**	**SO-III**	**SO-IV**	**PHY1**	**PHY2**	**PHY3**	**Syn03**
Hip	7.1	9.0	6.6	8.9	7.4	7.3	9.2	11.9
Knee	4.4	5.6	3.7	5.5	4.5	4.5	5.7	7.1
Ankle	8.4	8.1	8.0	8.8	8.5	8.5	8.1	11.5

Besides, the computational efficiency of the different approaches studied in this work is compared in Table 1. All calculations were performed on an Intel® Core™ i7-6700 K processor running at 4.00 GHz with 16 GB of RAM. With a mean computational time of 2.5 s to simulate a gait cycle, SO-I is the fastest approach, while PHY1 is the slowest one (225.2 s).

**Fig. 7:**
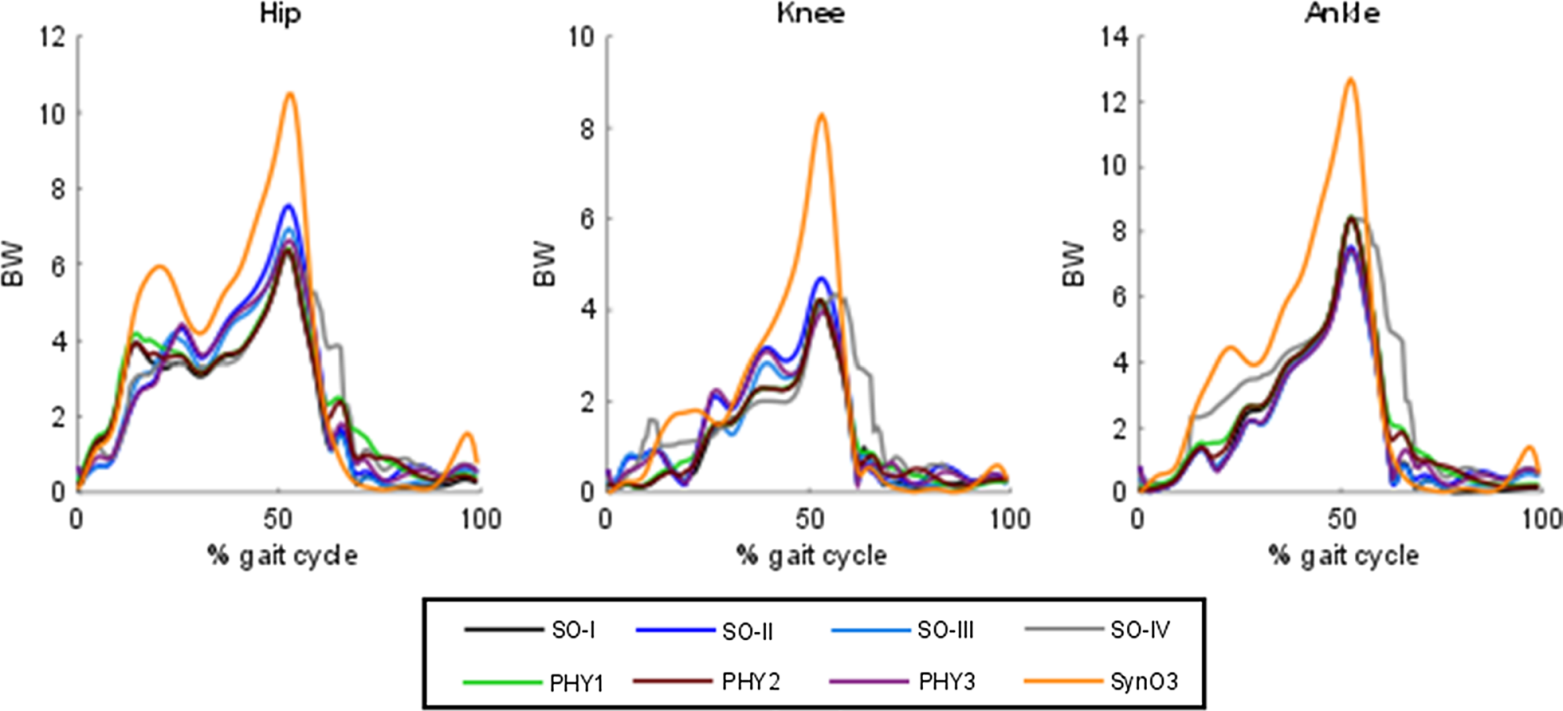
Joint reaction forces at hip, knee and ankle obtained with different muscle recruitment approaches for a healthy subject

Finally, the resulting joint reaction forces at hip, knee and ankle for the different muscle recruitment approaches are compared (Fig. 7 and Table 4). Similar joint reaction forces are obtained with the non-synergy-based approaches: SO-I, PHY1 and PHY2 offer almost the same results, while SO-II and PHY3 show joint reactions slightly higher than their counterparts at hip and knee levels. However, the joint reaction forces at hip, knee and ankle calculated from the muscle forces estimated with SynO3 are much higher than those obtained with the other approaches (Table 5).

## Discussion

This work offers a comparison of the efficiency and accuracy of: (i) four different criteria; (ii) three different physiological representations of the musculotendon actuator dynamics; (iii) a synergy-based method; all of them in the framework of inverse-dynamics based optimization. All the approaches were used under the same conditions by taking the same inputs from motion/force/EMG gait analyses performed on ten healthy subjects. Results obtained with the different methods do not present large discrepancies. Higher complexity of the method does not guarantee better results, as the best correlations with experimental values were obtained with two simplified approaches.

First, muscles activations obtained from SO and four different criteria exhibit visually different shapes along with some similarities. In addition, mean across subjects Pearson correlation coefficient *r* values between EMG vs. muscle activations of all the criteria do not present significant discrepancies. The best correlations were obtained with the simplest and fastest criterion (SO-I), which yielded a correlation of 74%, while the worst correlations were obtained with the most involved criterion (SO-IV).

Second, it was observed that the physiological representation of the musculotendon actuator dynamics does not affect the estimation of muscle forces during gait. Muscle activation shapes, experimental correlations and joint reaction forces are almost the same as those obtained through the non-physiological method (SO). The paired sampled t-tests demonstrated that SO-I, PHY1, PHY2 and PHY3 are statistically similar (p < 0.05). The same conclusion was drawn by De Groote et al. [15], Anderson and Pandy [6] and Millard et al. [7]. However, for faster, higher-powered tasks, like running or jumping, a compliant tendon model could be preferable. Moreover, despite its disadvantages (harder to implement and higher computational time), the physiological approach served to implement some Hill-based energy expenditure methods [47, 48], since it provides the muscular variables required as inputs. Despite the physiological realism of these approaches, it must be pointed out that the Hill-based muscle dynamics does not include the activation of gamma motor neurons and stretch receptors (i.e., proprioceptive receptors), which can induce minor activation in the stretched muscles during gait [49]. Consequently, some discrepancies can be explained by the EMG-linear envelope extraction procedure that may not properly demodulate neural excitations from the motor neurons action potentials, or because the noise contamination from cross-talk and movement artifacts, two intrinsic limitations in surface EMG measurements in addition of the inability to access deep muscles [23].

Third, as previously observed in [40], the synergy structure imposed within the SynO approach did not improve prediction of muscle activations during gait. This approach showed significant differences with the best approaches offered in this work (SO-I, PHY1, PHY2 and PHY3). The muscle synergy hypothesis has been notoriously difficult to prove or falsify [50], and results of this study do not allow to draw a conclusion in this regard. It can only be said that the SynO approach offers reasonable prediction of muscle activations and that its reduced dimensional control space could be beneficial for applications such as epidural electrical stimulation [51] or motion control and prediction [38].

Finally, all the estimated joint reaction forces at the hip were higher than the direct experimental measurements reported in the literature [52–54]. Brand et al. reported that hip contact-force predictions in the literature are higher than force measurements because of modeling assumptions [54]. In this work, and in the literature [9, 54], it has been shown that, paradoxically, physiological representation of the musculotendon actuator dynamics increases rather than reduces the discrepancies between force predictions and measurements, due to its constraints. Same conclusion can be drawn for the SynO approach, which disproportionately increases the joint forces due to its imposed synergy structure and reduced dimensional control space. Shourijeh and Fregly observed that the joint stiffness results were visibly different between the SynO and SO solutions, and that the stiffness decreased as the number of synergies was increased [13].

## Conclusion

In conclusion, this study evaluated several approaches to predict muscle activations during gait by comparing them with EMG measurements obtained experimentally, and found that higher complexity of the method does not guarantee better results. No significant differences among predicted EMG patterns within different physiological representations of the musculotendon were found. However, the simplified physiological approach with rigid tendon and activation time considered, presented the best accuracy and a very competitive computational time.

## Data Availability

The datasets generated for this study are available on request to the corresponding author.
